# Intraoperative Confocal Laser Endomicroscopy *Ex Vivo* Examination of Tissue Microstructure During Fluorescence-Guided Brain Tumor Surgery

**DOI:** 10.3389/fonc.2020.599250

**Published:** 2020-12-04

**Authors:** Evgenii Belykh, Xiaochun Zhao, Brandon Ngo, Dara S. Farhadi, Vadim A. Byvaltsev, Jennifer M. Eschbacher, Peter Nakaji, Mark C. Preul

**Affiliations:** ^1^ Department of Neurosurgery, The Loyal and Edith Davis Neurosurgical Research Laboratory, Barrow Neurological Institute, St. Joseph’s Hospital and Medical Center, Phoenix, AZ, United States; ^2^ Department of Neurosurgery and Innovative Medicine, Irkutsk State Medical University, Irkutsk, Russia; ^3^ Department of Neuropathology, Barrow Neurological Institute, St. Joseph’s Hospital and Medical Center, Phoenix, AZ, United States

**Keywords:** brain histology, brain tumor, confocal laser endomicroscopy, fluorescein sodium, fluorescence, frozen section, optical biopsy

## Abstract

**Background:**

Noninvasive intraoperative optical biopsy that provides real-time imaging of histoarchitectural (cell resolution) features of brain tumors, especially at the margin of invasive tumors, would be of great value. To assess clinical-grade confocal laser endomicroscopy (CLE) and to prepare for its use intraoperatively *in vivo*, we performed an assessment of CLE *ex vivo* imaging in brain lesions.

**Methods:**

Tissue samples from patients who underwent intracranial surgeries with fluorescein sodium (FNa)–based wide-field fluorescence guidance were acquired for immediate intraoperative *ex vivo* optical biopsies with CLE. Hematoxylin-eosin–stained frozen section analysis of the same specimens served as the gold standard for blinded neuropathology comparison. FNa 2 to 5 mg/kg was administered upon induction of anesthesia, and FNa 5 mg/kg was injected for CLE contrast improvement. Histologic features were identified, and the diagnostic accuracy of CLE was assessed.

**Results:**

Of 77 eligible patients, 47 patients with 122 biopsies were enrolled, including 32 patients with gliomas and 15 patients with other intracranial lesions. The positive predictive value of CLE optical biopsies was 97% for all specimens and 98% for gliomas. The specificity of CLE was 90% for all specimens and 94% for gliomas. The second FNa injection in seven patients, a mean of 2.6 h after the first injection, improved image quality and increased the percentage of accurately diagnosed images from 67% to 93%. Diagnostic CLE features of lesional glioma biopsies and normal brain were identified. Seventeen histologic features were identified.

**Conclusions:**

Results demonstrated high specificity and positive predictive value of *ex vivo* intraoperative CLE optical biopsies and justify an *in vivo* intraoperative trial. This new portable, noninvasive intraoperative imaging technique provides diagnostic features to discriminate lesional tissue with high specificity and is feasible for incorporation into the fluorescence-guided surgery workflow, particularly for patients with invasive brain tumors.

## Introduction

Complete resection within the bounds of functional safety is the goal of neurosurgical treatment for most brain tumors, especially invasive tumors, such as high-grade glioma (HGG) (1, 2). Of particular importance are intraoperative tools that aid not only in the identification of abnormal brain tissue that should be resected but also in its differentiation from healthy brain tissue that should be preserved (3). Here we report an investigation of a relatively new handheld cellular resolution imaging tool that may prove valuable for intraoperative noninvasive optical biopsy.

Previous generations of the confocal laser endomicroscope (CLE) demonstrated initial feasibility in visualizing brain tumor microstructure with significant diagnostic accuracy (4). However, these designs were largely based on an imaging platform directly adapted from those used for gastrointestinal imaging. Thus, the US Food and Drug Administration (FDA) approved the use of these systems in the brain only for experimental purposes, and previous generations of CLE were used in only three limited trials that included *in vivo* and *ex vivo* intraoperative use (5–7). Disadvantages of the early designs included the necessity to sterilize the scanning probe between procedures; the wide 7-mm diameter of the probe, which limited the use of the CLE within narrow surgical corridors; and the straight profile of the probe as it fit into the surgeon’s hand.

The novel-generation commercial clinical grade CLE was developed to address these shortcomings. The FDA-approved CLE system has a 5-mm-diameter probe that is curved like an aspiration instrument, and it has increased imaging resolution and is designed to work with a removable sterile sheet. Since the use of CLE for visualization of brain tumors was first reported, wide-field fluorescence guidance surgery has increased in popularity. Thus, fluorescein sodium (FNa) began to be administered as part of the standard neurosurgical procedure so that the bright glow of FNa fluorescence could be visualized with an operating microscope used in the Yellow560 mode.

To assess the clinical grade CLE and prepare for its intraoperative use *in vivo*, we designed an assessment of its *ex vivo* imaging performance in brain lesions, particularly invasive brain tumors. We studied CLE use during routine fluorescence-guided surgery (FGS) with the operating microscope to visualize and compare CLE tissue-imaging microstructure in *ex vivo* samples with frozen section biopsies of brain tumors. We assessed the feasibility and diagnostic accuracy of CLE optical biopsies of brain lesions to identify relevant practical and methodologic variables for future *in vivo* use in clinical studies.

## Materials and Methods

### Study Design

This prospective study was conducted at St. Joseph’s Hospital and Medical Center with approval from the Institutional Review Board for Human Research (IRB No, 10BN130). All participants gave informed voluntary consent for participation in this study and were recruited from patients who were operated on from August 2016 to May 2019. Patients eligible for inclusion were adults (>18 years old) scheduled at our institution for neurosurgical lesion removal involving FNa contrast administration. This convenience sample of patients with brain lesions, mostly tumors, were enrolled whenever there was availability of CLE for operations conducted by the neurosurgeons participating in the study. Standard intraoperative techniques were used during tumor resection, including neuronavigation, operating microscope, endoscopic assistance, and, in some patients, intraoperative functional mapping. Deidentified brain tumor samples were used for this study. Demographic characteristics were deemed irrelevant to the optical biopsy interpretation and quality-assessment aspects of this study and were not recorded.

### FNa Administration

FNa was administered intravenously at the induction of anesthesia to create a contrast for wide-field fluorescence guidance using the Yellow560 mode of the operating microscope. The FNa dose was determined by the operating neurosurgeon. FNa 2 mg/kg was used in patients with gliomas and meningiomas, and FNa 5 mg/kg was used in patients with metastatic lesions. FNa was readministered at a 5-mg/kg dose if deemed necessary by the neurosurgeon. One patient received 40 mg/kg of FNa at the induction of anesthesia only.

### Tissue Collection and Processing

Tissue samples that were removed as a part of the standard neurosurgical procedure were obtained during the tumor resection for examination. These samples were separate from those obtained for clinical care (i.e., frozen-section and permanent-section analyses). When possible, multiple biopsies of the tumor core and tumor bed area were acquired. The number of biopsies was determined by the operating neurosurgeon.

Samples were placed on a moisturized nonadherent surgical dressing, transferred to the CLE station (CONVIVO, Carl Zeiss Meditec, AG, Jena, Germany) in the same operating room, and imaged using the CLE probe affixed in a probe holder in an upright position. Tissue samples were imaged with CLE immediately after acquisition from the brain. The tissue samples were gently placed with microsurgical forceps to obtain optimal imaging. To obtain optimally balanced CLE images, we used a 488 nm excitation laser and a 517.5. to 572.5-nm bandpass filter at 1× zoom with automatic gain, resulting in resolution of 1920 × 1080 pixels and a 267 × 475 μm field of view.

The neurosurgeon was able to review the CLE images during the operation because they were displayed intraoperatively in real time on a large LED screen. When image brightness was not sufficient, the FNa was readministered according to the prescribed dosage at the request of the neurosurgeon.

After CLE imaging, tissue samples were placed on a piece of moistened absorbent cotton material in a plastic container. Samples were then submitted for histologic processing and hematoxylin-eosin (H&E) staining in the neuropathology department.

### Image Analysis

The collected CLE images were processed using FIJI open-source software by applying the “despeckle” filter and creating short videos (3–20 frames in length) of select imaging locations. Deidentified CLE optical biopsies and H&E-stained sections were reviewed retrospectively in a blinded fashion by a board-certified neuropathologist (J.E.) competent in interpreting CLE images. The neuropathologist had no clinical information except that the biopsy had been performed during an intracranial procedure for removal of a lesion.

CLE optical biopsies were presented as multiple still images and video-like image loops. A CLE optical biopsy was graded as “lesional” when it was deemed representative of a tumor or necrotic tissue; as “normal” when it was representative of normal or reactive brain tissue; and as “nondiagnostic” when it was not representative of tumor or necrotic tissue (i.e., nondiagnostic of tumor). Seventeen histologic features were identified and assessed as “present” or “absent” in each CLE biopsy. The overall quality of CLE images for each optical biopsy was graded subjectively on a 6-point scale of zero to 5: 0 (not diagnostic or very bad), 1 (bad), 2 (poor), 3 (average), 4 (good), and 5 (very good). This quality assessment based on a subjective interpretation of clarity and brightness of tissue microstructure was performed by a neurosurgeon and a neuropathologist experienced in CLE and other fluorescence cell-imaging technologies.

H&E slides of biopsies were graded as “lesional” when they were representative of tumor tissue useful for diagnosis or interpretations and as “nonlesional” when they were representative of nontumor tissue, such as normal brain or gliotic reactive brain tissue. All H&E slides were useful for such interpretations. Each H&E slide was also labeled on the basis of the most likely diagnosis for a particular slide or as “nondiagnostic.”

### Image Interpretation

To assess how experience in working with CLE affects the interpretation of CLE images, we assigned two additional appraisers (both experienced senior neurosurgeons) to review a set of CLE images for interpretation. The first neurosurgeon (M.C.P.) was experienced in interpreting CLE images but was blinded to the image acquisition process. The second neurosurgeon (V.A.B.) had limited experience in interpreting CLE images but had assistance from a general pathologist who also lacked experience in interpreting CLE images. Prior to assessment, both neurosurgeons were instructed using a separate set of images on the key histologic features identifiable on CLE images. No information was provided to the neurosurgeons or general pathologist regarding case history, imaging, surgical information, or diagnosis.

### Statistical Analysis

Statistical analysis was performed in Excel (Microsoft, Inc., Redmond, WA). The interpretation of CLE images and H&E histologic sections was compared. We also assessed CLE image quality and timing and dose of FNa injection. Exploratory diagnostic accuracy was calculated using a 2×2 table and standard formulas for the sensitivity, specificity, positive predictive value, and negative predictive value; results were reported according to the Standards for Reporting Diagnostic Accuracy Studies guidelines (8). For statistical analysis, CLE optical biopsies that were nondiagnostic of tumor tissue or that were labeled as normal tissue were used as a negative CLE test result. For the purpose of analysis, inconclusive H&E results were treated as “nonlesional.”

## Results

### Descriptive Analysis

**Figure 1:**
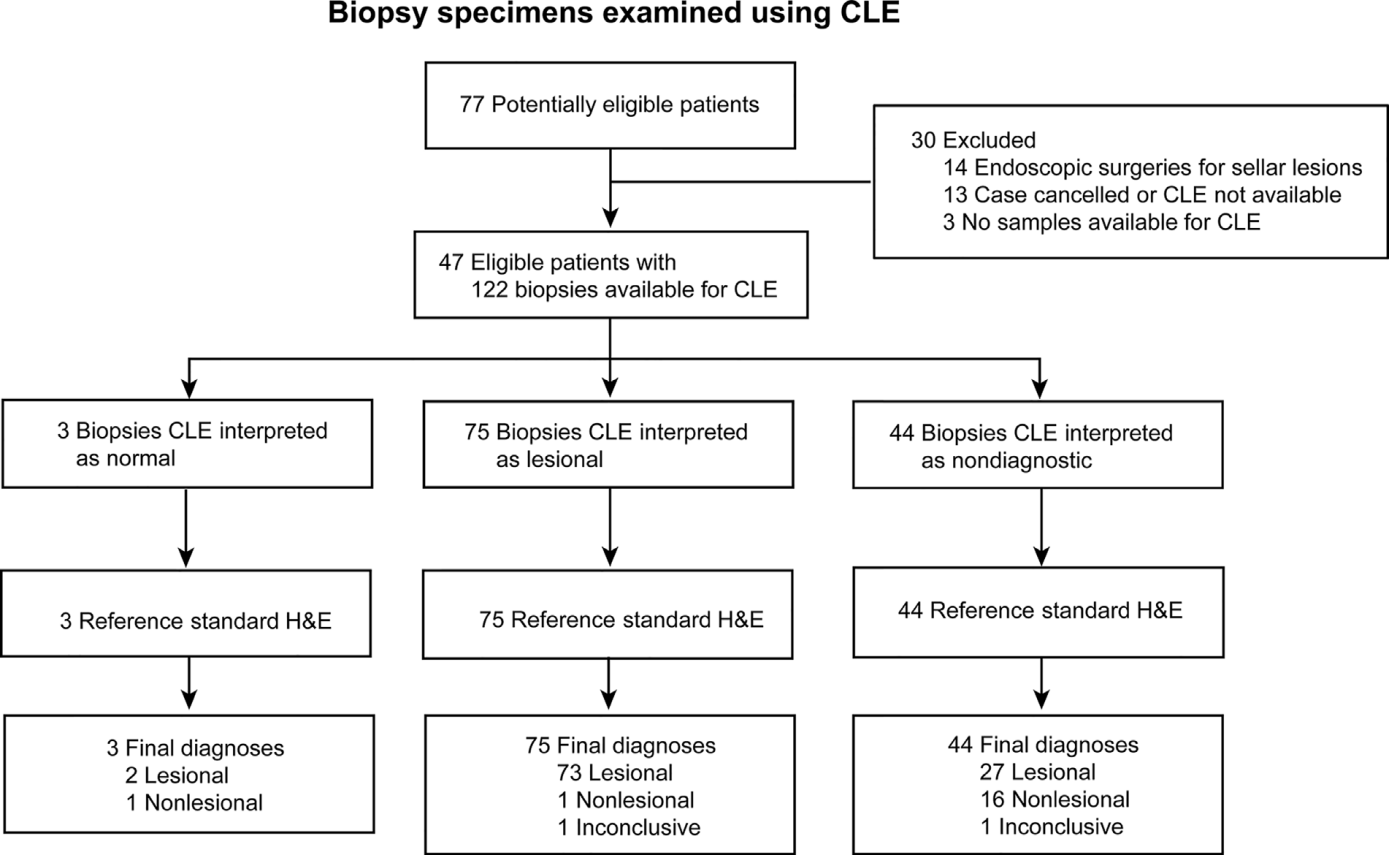
Flowchart of 122 biopsy specimens from 47 patients with brain tumors examined using confocal laser endomicroscopy (CLE). H&E, hematoxylin-eosin. *Used with permission from Barrow Neurological Institute, Phoenix, Arizona*.

Seventy-seven potentially eligible patients provided consent during the study period. CLE imaging was not performed for 13 patients because of surgery cancelation or CLE unavailability. Fourteen patients were excluded because their procedures were endoscopic transsphenoidal surgeries, and three patients were excluded because no biopsy samples were available for analysis. Thus, CLE imaging was available for 47 patients with 122 matched optical and histologic biopsies available for analysis (**Table 1**, **Figure 1**). Preliminary diagnoses by tumor type were as follows: 32 gliomas (19 primary glioblastomas, 5 recurrent glioblastomas, 5 infiltrating gliomas, 3 low-grade gliomas), 7 meningiomas, 4 metastatic brain lesions, 1 choroid plexus carcinoma, 1 craniopharyngioma, 1 schwannoma, and 1 arteriovenous malformation (reactive normal brain). All specimens were successfully analyzed *ex vivo* with CLE immediately after acquisition.

**Table 1:** General characteristics on confocal laser endomicroscopy of 122 biopsy specimens from 47 cases.

Final diagnosis	Cases	Biopsies	Biopsies per case, mean	Diagnosis on the basis of H&E staining (no. of biopsies)
GBM	19	52	2.7	HGG (30); infiltrating glioma (12); reactive gliotic brain (5); normal brain (3); necrosis (1); inconclusive (1)
Recurrent GBM	5	24	4.8	HGG (11); infiltrating glioma (10); necrosis (1); reactive gliotic brain (1); inconclusive (1)
Infiltrating glioma	5	13	2.6	Infiltrating glioma (11); reactive gliotic brain (1); normal brain (1)
LGG	3	8	2.7	Infiltrating glioma (4); normal brain (4)
Metastasis	4	9	2.3	Metastasis (8); reactive gliotic brain (1)
Meningioma	7	9	1.3	Meningioma (9)
Choroid plexus carcinoma	1	4	4	Choroid plexus carcinoma (4)
Craniopharyngioma	1	1	1	Craniopharyngioma (1)
Schwannoma	1	1	1	Schwannoma (1)
AVM	1	1	1	Reactive gliotic brain (1)

AVM, arteriovenous malformation; GBM, glioblastoma multiforme; H&E, hematoxylin-eosin; HGG, high-grade glioma; LGG, low-grade glioma.

### Second Dose of FNa

Resection of the lesion was completed in all patients under operating microscope visualization with and without Yellow560 mode irrespective of regard for *ex vivo* CLE analysis. In 7 cases (5 gliomas, 1 metastasis, and 1 choroid plexus carcinoma), FNa (5 mg/kg) was subsequently administered after the initial resection to evaluate the edge with subsequent biopsy acquisition for CLE analysis. FNa was readministered at a mean (SD) of 157 (52) min after the first administration. Analysis of these specimens showed improved brightness and contrast and improved overall image quality. For the cases in which the reinjection was performed, the percentage of images with an accurate diagnosis increased from 67% (18 of 27) to 93% (14 of 15), and the percentage of nondiagnostic CLE decreased from 26% (7 of 27) to 13% (2 of 15), although our study was not powered to detect differences in these subgroups (P=0.11 and P=0.31, respectively, by χ^2^ analysis).

### Timing of FNa Injection

In many cases, biopsy acquisition occurs more than 90 min after the first FNa administration, which results in suboptimal contrast in CLE images (7). This decrease in image quality was also found for biopsies in the current study when the FNa was injected 1 to 5 min before imaging. However, the analysis of all biopsies, as well as the glioma-only biopsies obtained at different time points after FNa injection, showed no correlation between the timing of FNa administration and image quality (r=−0.14, P>0.05) or with the probability of the CLE optical biopsy being used to diagnose specimens as either lesional or nondiagnostic (logistic regression P=0.93).

### CLE Histologic Features

**Figure 2:**
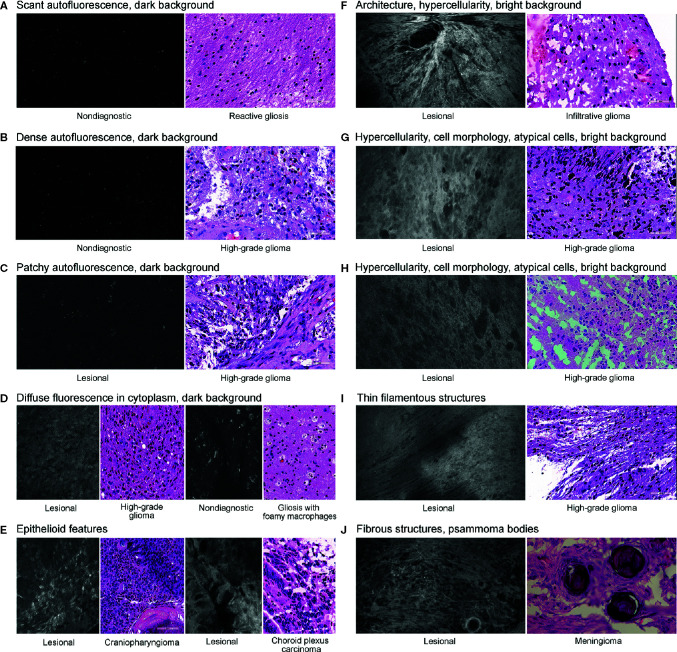
Histologic features identified on confocal laser endomicroscopy images of various brain tumors and matching hematoxylin-eosin images. We identified three types of autofluorescence: scant **(A)**, dense **(B)**, and patchy **(C)**. We also identified the following image characteristics as separate histologic features: diffuse intracytoplasmic fluorescence **(D)**, epithelioid features **(E)**, hypercellularity **(F–H)**, filamentous structures **(I)**, and fibrous structures **(J)**. An example of characteristic identifiable tissue architecture as a histologic feature is shown in **(F)**. Examples of characteristic identifiable features of cell morphology and atypical cells are shown in **(G)** and **(H)**. An example of psammoma bodies is presented in **(J)**. We classified the brightness of background (extracellular space) as dark **(A**–**D)** or bright **(F**–**I)**. *Used with permission from Barrow Neurological Institute, Phoenix, Arizona*.

**Figure 3:**
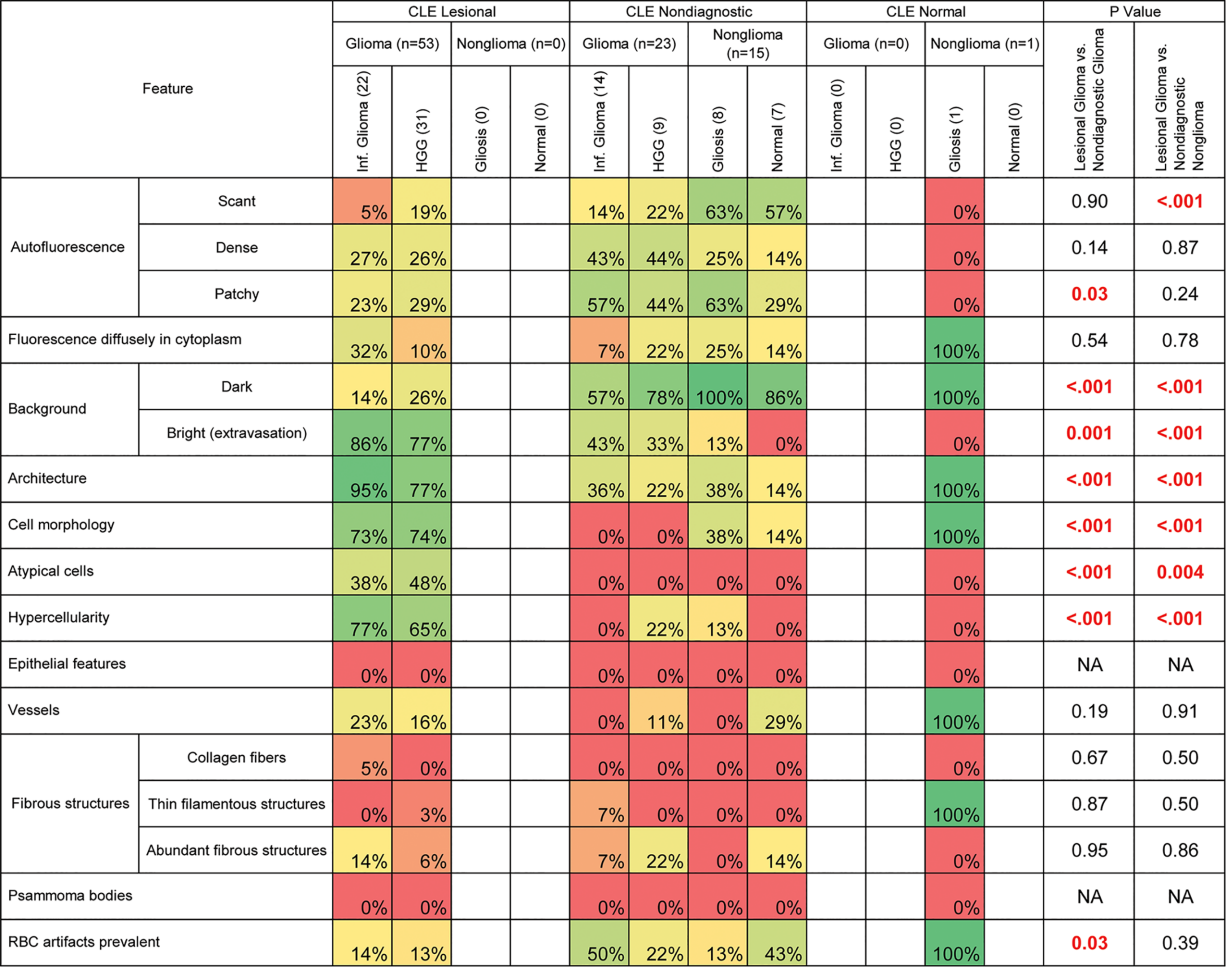
Prevalence of histologic features among gliomas and normal or reactive brain tissue specimens. This analysis excluded 2 confocal laser endomicroscopy (CLE) optical biopsies with false-positive results, 2 CLE optical biopsies with false-negative results, and 1 nondiagnostic sample stained with hematoxylin-eosin. Table cells are shaded to reflect the prevalence of the specified feature among each group of specimens on a spectrum from red (0%) to green (100%). Boldface type indicates statistical significance. HGG, high-grade glioma; NA, not available; RBC, red blood cell. *Used with permission from Barrow Neurological Institute, Phoenix, Arizona*.

Various histologic features were assessed to elucidate characteristics of diagnostic and nondiagnostic CLE optical biopsies (**Figure 2**). The prevalence of identifiable histologic features on CLE imaging was specifically studied in select biopsies of glioma, reactive gliosis, and normal brain samples (**Figure 3**).

#### Autofluorescence

Three types of autofluorescence were distinguished: scant, patchy, and dense. Scant autofluorescence was frequently observed in nonglioma samples that were deemed nondiagnostic by CLE (P<0.001), but there were no significant differences in the frequencies of patchy and dense fluorescence.

#### Diffuse Intracellular Fluorescence

In some CLE optical biopsies, cells exhibited diffuse accumulation of FNa in the cytoplasm. This accumulation was especially noticeable in tissue from carcinomas. In gliomas, however, the frequency of this pattern was not significantly different among nontumor and glioma biopsies.

#### Background Color

The pattern of CLE images in which the extracellular space was dark, and usually darker than the cells, was significantly more prevalent in nonglioma specimens (P<0.001) and in glioma specimens that were rated nondiagnostic (P<0.001). However, bright background was observed in most glioma samples diagnosed as lesional, and it was almost absent in biopsies from normal and reactive brain (P<0.001).

#### Cellular Features

Features such as tissue architecture, cell morphology, atypical cells, and hypercellularity were more prevalent in lesional glioma biopsies than in normal and reactive brain (P<0.001 for all). These features were also more prevalent in lesional glioma biopsies than in glioma biopsies that were labeled nondiagnostic on CLE (P<0.001 for all).

#### Epithelial Features

The pattern of hypercellular areas with cells contacting each other and having glandular or epithelial arrangement was observed in all three metastases, in one choroid carcinoma, and in the epithelial portion of the craniopharyngioma specimen. This feature was not observed in gliomas.

#### Vessels

Vessels were not frequently identified, most likely because of the lack of blood flow in *ex vivo* samples, which hampered detection. No differences were observed in their frequency of occurrence in the diagnostic, nondiagnostic, and normal brain samples. However, normal capillaries with FNa-stained vessel walls were deemed more characteristic of normal brain, or possibly of invasive glioma, than of cellular tumor.

#### Fibrous Structures

Various acellular fibrous structures were encountered, mostly in meningiomas, but were rarely seen in gliomas. However, some thin filamentous structures representing myelinated cell processes could be seen in gliomas. Psammoma bodies were identified in meningiomas as large, round, contrast-impermeable structures.

#### Red Blood Cell Artifacts

Although red blood cell artifacts are inevitable during CLE imaging, an abundance of blood was encountered significantly more often in nondiagnostic CLE biopsies of gliomas than in lesional CLE biopsies of gliomas (P=0.02).

### Blinded Review by Trained Neuropathologist

**Figure 4:**
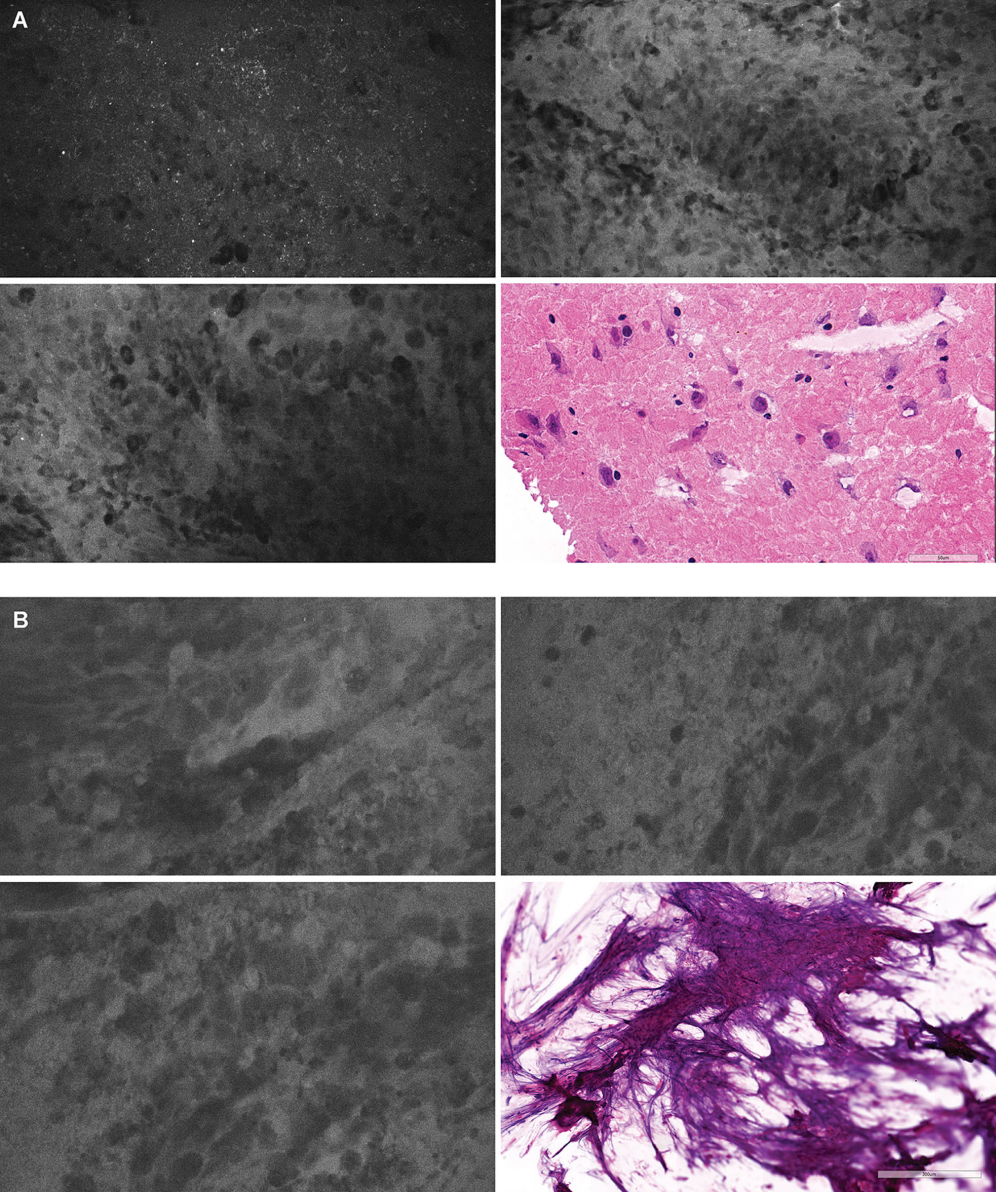
Positive confocal laser endomicroscopy (CLE) optical biopsies with negative hematoxylin-eosin (H&E) stain results. **(A)** In one optical biopsy, extravasation of FNa highlighted the background and revealed hypercellular areas of atypical large cells. Overall, this CLE optical biopsy was thought to be highly representative of a lesional tissue. However, the H&E-stained specimen was read as normal cortex without a tumor. We hypothesized that there might be a sampling error and that the specimen contained a tumor area that had been missed during histologic sectioning. **(B)** This optical biopsy demonstrated extravasation of FNa with hypercellular areas containing large atypical cells highly suggestive of a cellular tumor. However, because of the scant tissue available for histologic processing, the H&E stain results were inconclusive. Both H&E-stained specimens were treated as nonlesional for diagnostic accuracy analysis. However, if these two H&E biopsy specimens had been treated as lesional, then both specificity and positive predictive value would be 100%. *Used with permission from Barrow Neurological Institute, Phoenix, Arizona*.

The overall diagnostic accuracy for all biopsies analyzed by a trained neuropathologist was 75%. Lesional CLE biopsies were used as positive CLE test results; nonlesional CLE, which included nondiagnostic and true negative normal or reactive brain CLE, were used as negative CLE test results (**Table 2**). Diagnostic accuracy in various tumor types was elucidated by performing subgroup analyses in glioma, metastases, and meningioma samples, which confirmed high positive predictive value of CLE optical biopsies (98%, 91%, and 83%, respectively) (**Table 3**). Notably, two CLE optical biopsies that were labeled as lesional had nonlesional H&E labeling such that they might have been true positives, which would further improve specificity and positive predictive value to 100% for all specimens and for gliomas (**Figure 4**).

**Table 2:** Confocal laser endomicroscopy interpretation of 122 biopsy specimens from 47 patients.

H&E Diagnosis	Specimens	Confocal Laser Endomicroscopy Interpretation				
		Lesional		Nonlesional		
				Normal		Nondiagnostic
		TP	FP	TN	FN	
Choroid plexus carcinoma	4	3				1
Craniopharyngioma	1	1				
HGG	41	31			1	9
Infiltrating glioma	37	22			1	14
Meningioma	9	6				3
Metastasis	8	7				1
Necrosis	2	2				
Normal brain	8		1			7
Reactive gliotic brain	9			1		8
Schwannoma	1	1				
Inconclusive	2	1				1

H&E, hematoxylin-eosin; HGG, high-grade glioma: FN, false negative; FP, false positive; TN, true negative; TP, true positive.

**Table 3:** Diagnostic accuracy of *ex vivo* confocal laser endomicroscopy (CLE) analyzed by a neuropathologist experienced in CLE image interpretation.

Value	All samples	Gliomas	Carcinomas^a^	Meningiomas
Sensitivity	72 (62–80)	66 (55–76)	83 (51–97)	63 (26–90)
Specificity	90 (67–98)	94 (69–100)	94 (69–100)	94 (69–100)
Positive predictive value	97 (90–100)	98 (89–100)	91 (57–100)	83 (36–99)
Negative predictive value	38 (25–54)	37 (23–53)	89 (64–98)	84 (60–96)

Data are reported as percentage (95% confidence interval).

^a^Carcinomas included metastatic lesions and choroid plexus carcinomas.

### Blinded Review by Trained and Untrained Appraisers

**Figure 5:**
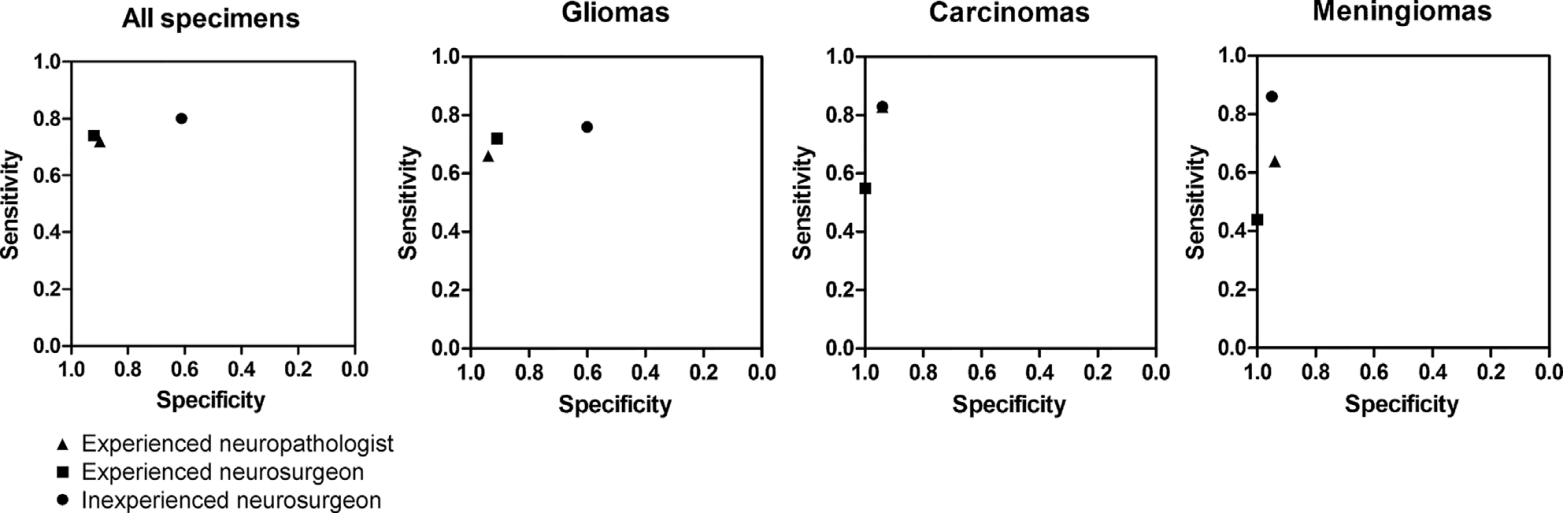
Diagnostic accuracy diagrams for all specimens together and for gliomas, carcinomas, and meningiomas analyzed separately, as assessed by an experienced neuropathologist, an experienced neurosurgeon, and an inexperienced neurosurgeon using confocal laser endomicroscopy. *Used with permission from Barrow Neurological Institute, Phoenix, Arizona*.

Overall diagnostic accuracy for all biopsies blinded for analysis by a neurosurgeon with experience interpreting CLE images was 78% vs 71% for the neurosurgeon without CLE image-reading experience. The comparative diagnostic accuracy data are presented in **Figure 5** and **Table 4**. Both the experienced neuropathologist and the experienced neurosurgeon demonstrated higher specificity and positive predictive value for gliomas.

**Table 4:** Diagnostic accuracy of *ex vivo* confocal laser endomicroscopy analyzed by an experienced neuropathologist, an experienced neurosurgeon, and an inexperienced neurosurgeon.

Tissue type, diagnostic parameter	Experienced neuropathologist	Experienced neurosurgeon	Inexperienced neurosurgeon
All samples			
Sensitivity	72 (62–80)	74 (65–83)	80 (70–90)
Specificity	90 (67–98)	92 (69–98)	61 (44–72)
Positive predictive value	97 (90–100)	97 (90–100)	71 (59–80)
Negative predictive value	38 (25–54)	47 (31–61)	72 (56–84)
Glioma			
Sensitivity	66 (55–76)	72 (61–82)	76 (62–87)
Specificity	94 (69–100)	91 (67–98)	60 (42–72)
Positive predictive value	98 (89–100)	96 (87–99)	66 (52–78)
Negative predictive value	37 (23–53)	49 (31–69)	71 (53–84)
Carcinoma^a^			
Sensitivity	83 (51–97)	55 (32–76)	83 (51–97)
Specificity	94 (69–100)	100 (60–100)	94 (68–100)
Positive predictive value	91 (57–100)	100 (68–100)	91 (57–100)
Negative predictive value	89 (64–98)	50 (24–71)	89 (62–98)
Meningioma			
Sensitivity	63 (26–90)	44 (21–69)	86 (42–99)
Specificity	94 (69–100)	100 (63–100)	95 (71–100)
Positive predictive value	83 (36–99)	100 (56–100)	86 (42–99)
Negative predictive value	84 (60–96)	53 (27–73)	95 (71–100)

Data are reported as percentage (95% confidence interval).

^a^Includes metastatic lesions and choroid plexus carcinoma.

### Adverse Effects

No adverse effects due to biopsy acquisition or FNa administration were noted. One patient was inadvertently administered 40 mg/kg of FNa at the induction of anesthesia, resulting in yellowish skin discoloration that resolved uneventfully within 48 h. No other adverse reactions were observed.

## Discussion

The projected clinical applications for the use of CLE in neurosurgery are to guide biopsy acquisition, to provide preliminary diagnosis in a manner similar to that of frozen section, and to perform screening for tumor detection during resection. In this study, we evaluated CLE optical biopsies of 122 *ex vivo* brain samples from 47 patients. The results demonstrated high sensitivity for this diagnostic technology in detecting cellular brain tumor tissue, which has significantly informative clinical value.

### Image Interpretation

Our results demonstrated that a neuropathologist and neurosurgeons experienced in reading CLE images interpreted images of gliomas with greater specificity than an inexperienced neurosurgeon, even in consultation with an inexperienced pathologist. Previous reports have indicated that special training and experience are necessary to interpret the gray scale confocal images of intravital microscopy (9–13). However, in our study, the scores are not greatly dissimilar among the experienced neuropathologist, the experienced neurosurgeon, and the inexperienced neurosurgeon assisted by a general pathologist. The interpretative accuracy of these scores is surprising, given that no information was provided to the personnel about the clinical or historical nature of the cases. In the normal surgery-pathology workflow, such additional contextual information would be a requisite part of the interpretation of CLE images for diagnosis and surgical guidance (7).

In the future, CLE image interpretation could be augmented by robust automated computer image analysis, which would allow for rapid identification of useful information from among the hundreds to thousands of images that may be acquired from one patient (14–16) and by digital telepathology consultation provided by an expert (17). In addition to confirming the diagnostic value of the new-generation CLE system, this study addresses the implementation of CLE into the surgery-pathology operating room workflow during FGS of the brain. As discussed below, the results provide data that will be helpful in planning future *in vivo* CLE studies in various tumor types.

### Implications for Practice: Incorporation of CLE into the Workflow of Fluorescence-Guided Brain Surgery

Building on a previous study in which FNa was administered approximately 5 min before CLE imaging (7), this study demonstrated the feasibility of using CLE in Yellow560 mode during FGS when FNa is administered upon induction of anesthesia. This timing of FNa injection favors navigation using the wide-field fluorescence operative microscope (18, 19), and it has been studied in several clinical trials (20, 21). However, after early administration of FNa, we found that the contrast in CLE images was insufficient in some cases, thus requiring readministration of FNa. Readministration of FNa resulted in improved CLE image quality. Interestingly, we found no correlation between the time of contrast injection and overall image quality, which may be related to the heterogeneity of the tumor tissue or blood artifacts and to the *ex vivo* nature of the study.

Reinjection of FNa showed important benefits for image quality and interpretation. Analysis of these specimens showed improved brightness, contrast, and improved overall image quality. For the cases in which the reinjection was performed, the percentage of images with an accurate diagnosis increased by 50%, and the percentage of nondiagnostic CLE decreased by 50%. In some cases, reinjection allowed us to obtain meaningful interpretation where with only a single injection at the start of the case, this would not have been possible. Although reinjection of FNa improved CLE image quality, the diffusion of FNa at the resection cavity can hamper the selectivity of Yellow560 wide-field fluorescence guidance, but this hindrance was not a major problem for us. However, there are some relevant considerations for reinjection of FNa. When administered later during surgery FNa extravasates at the regions of surgical injury creating false-positive fluorescence, which was the major critique of FNa as a contrast agent for wide-field fluorescence guidance (22). Future *in vivo* studies should address this problem. Most likely, CLE could best be used after resection under Yellow560 guidance to facilitate inspection of the surgical resection bed at the conclusion of surgery (i.e., to interrogate suspected tumor invasion into eloquent or surrounding cortex). Interestingly, when 40 mg/kg FNa was administered, CLE demonstrated high-quality images with excellent contrast in visualizing tumor cells, further supporting our approach to reinject FNa to increase image contrast and clarity.

Alternatively, FNa could be used for CLE optical biopsy during or at the completion of resection under 5-aminolevulinic acid (5-ALA) guidance. Simultaneous use of 5-ALA and FNa has been reported previously (23). Our experience with the new operating microscope in Blue400 and Yellow560 modes demonstrates that the two channels can be alternated without compromising image quality. Concurrent FNa-contrasted CLE may be advantageous during surgeries performed using near-infrared guidance (e.g., using indocyanine green [ICG]) because of the absence of spectral overlap with FNa (24, 25). Confocal-assisted fluorescence microscopy with ICG as a contrast has also been reported (26, 27). In view of future incorporation of CLE into the workflow of FGS, diagnostic accuracy using CLE should be compared to wide-field fluorescence imaging technology using various diagnostic fluorophore agents, such as FNa, 5-ALA, and ICG.

For safe maximal resection, other techniques should also be considered, particularly intraoperative stimulation and awake brain mapping using fluorescence (28). These and other functional methods are designed to be highly sensitive for eloquent brain areas that should be preserved, whereas the goal of FGS and CLE is to specifically detect and reveal tumor regions for the surgical decision on whether to resect. These two methods can therefore provide mutually complementary information.

The use of CLE requires coordination with the anesthesiologist for timely and accurate administration of the FNa dose. Importantly, we believe that close association with a neuropathologist, either remotely or in the operating room, is necessary, especially at the initial adoption stage of the CLE technology, as was the case for this *ex vivo* application. Coordination and communication between the neurosurgeon in the operating room using the CLE and the neuropathologist providing rapid examination of the images acquired on the fly is fully supported by this CLE technology, and this will be especially advantageous for *in vivo* use. The images can be ported to any computer workstation or even handheld computing device (e.g., “smart phone”) supporting image display. CLE systems will require and foster a close partnership and collaboration between the neurosurgeon and neuropathologist.

### Diagnostic Accuracy in Different Tumor Types

Because glioma tissue is often challenging to distinguish from normal brain tissue without special or enhanced imaging techniques, *in vivo* CLE optical biopsy of gliomas is of particular interest because it yields histopathologic imaging results immediately. With our experience from experimental animal studies and early clinical studies, we have identified characteristic CLE patterns and have developed confidence in our interpretation of CLE images and in our successful identification of hypercellular HGG samples (5, 29, 30). In contrast to previous studies, this study assessed multiple matched biopsies from the same patient, including multiple samples that were read as gliotic brain without tumor.

CLE images from within a tumor mass may readily display characteristic cellular features that are unique to and therefore diagnostic of the tumor type. However, although imaging within a main tumor mass may be useful for rapid histologic recognition and confirmation, the main purpose and advantage of the CLE is to recognize abnormal histologic features at the marginal regions of tumor to identify regions that can be surgically optimized. Such an imaging situation occurs within the surgical resection bed when assessing whether to extend the tumor removal. The CLE field of view is small (267 × 475 μm), and thus the concentration on identifiable histologic features at the tumor or pathological margins may not include readily visible diagnostic features that inform an exact tumor type. Instead, the focus should be on distinguishing abnormal histoarchitectural features, such as those described in the results.

**Figure 6:**
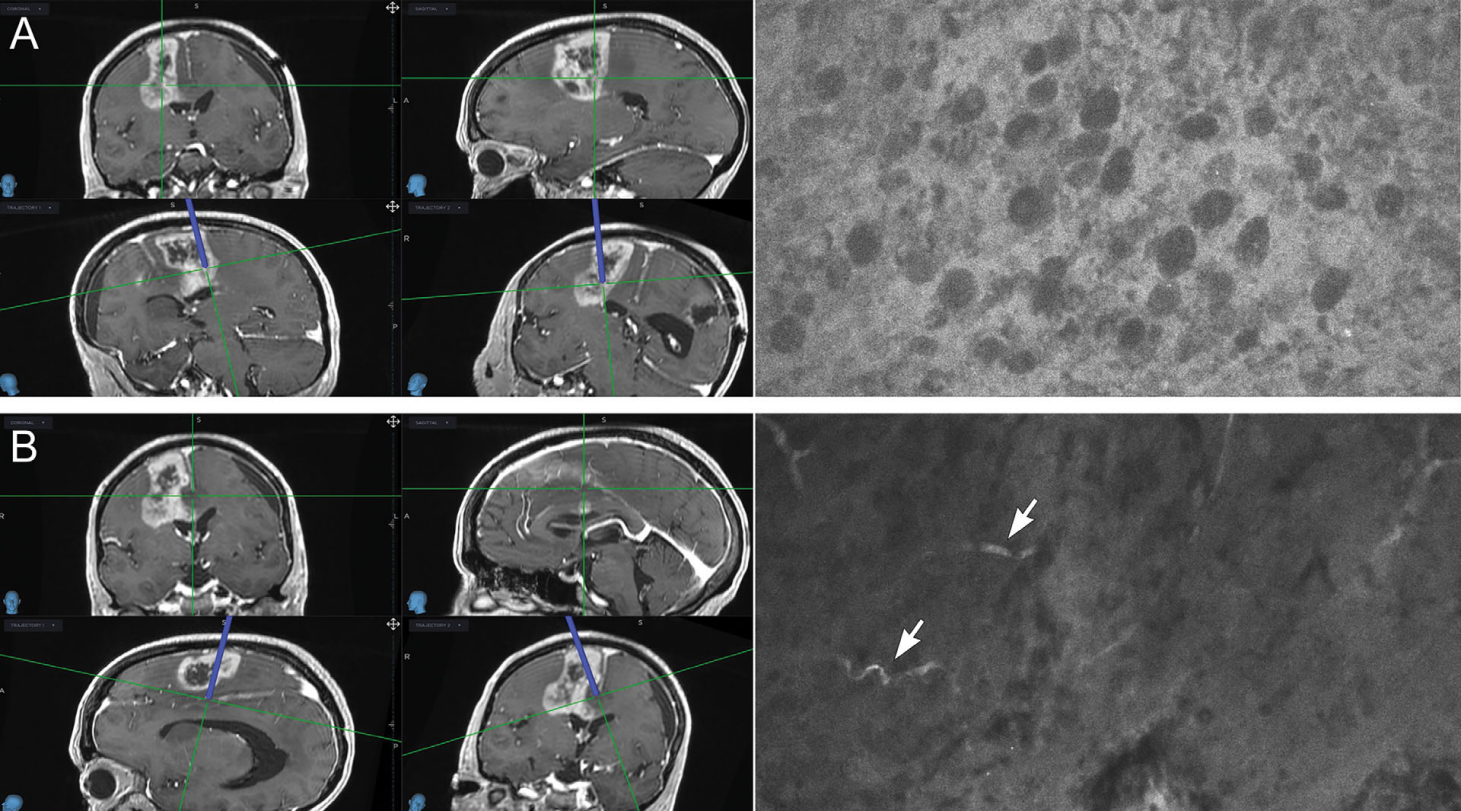
Illustrative example of confocal laser endomicroscopy (CLE) (*right*) used in combination with intraoperative navigation (*left*). Multiple biopsies were performed during resection of a high-grade glioma; two biopsies are shown. **(A)** A biopsy specimen obtained from a deep posterior contrast-enhancing tumor margin. CLE findings were positive for tumor showing atypical cellular features and hypercellularity. A diagnosis of high-grade glioma was made on the basis of hematoxylin-eosin (H&E) staining of this biopsy specimen. Resection was extended further and deeper. **(B)** A biopsy specimen was obtained from a deep noncontrast-enhancing posteromedial tumor margin. CLE had findings negative for tumor and showed normal-appearing vessels (*arrows*). This biopsy specimen was identified as normal cortex with focal vessels on H&E staining. *Used with permission from Barrow Neurological Institute, Phoenix, Arizona*.

Coupled with our previous studies on the differentiation of glioma tissue from injured normal brain in the murine model (31), the current work deepens our understanding of the CLE features of nontumor normal and gliotic brain tissue in humans. Most of the specimens of gliotic and normal brain tissue were labeled as nondiagnostic of brain tumor because they lacked typical cellular features and high cellularity, although most of them had dark extracellular backgrounds and some autofluorescent speckles. Normal cells were not clearly visible on these images, which impeded a definitive conclusion that the optical biopsy was representative of normal brain tissue. Interestingly, increased speckles on CLE images obtained with a similar device have previously been observed in low-grade gliomas after administration of 5-ALA (32), but they most likely represented autofluorescence (33). Whether this observation indicates the normal appearance of brain tissue using CLE imaging with FNa contrast is not clear. We believe that future *in vivo* studies should focus on the assessment of marginal tissue to better distinguish normal brain tissue when interpreting a dark CLE optical biopsy with autofluorescence in the absence of cellular architecture features (**Figure 6**).

With respect to metastatic lesions and choroid plexus carcinomas, CLE provides high-contrast, clear, and reliable images of cellular features that are highly diagnostic of cellular tumor. Most nondiagnostic CLE optical biopsies are attributable to blood artifacts that hide the actual tissue or the FNa contrast redistribution over time, which diminishes contrast among cells and among their nuclei, cytoplasm, and extracellular compartments.

In all available meningioma samples, including one composed of 80% dura and 20% invasive meningioma tissue, CLE provided clear high-contrast images of hypercellular tissue with fibrous structures and psammoma bodies, when present. Because normal marginal nonmeningioma samples were not available for CLE assessment, we used normal and gliotic brain tissue as negative controls for calculating the diagnostic accuracy in meningiomas. However, for future *in vivo* studies of meningiomas, multiple biopsies of the dura at the edge of the tumor should be obtained, similar to the tissue acquisition workflow in gliomas. We have thoroughly examined features of normal dura with FNa in a pig model (unpublished data), but its appearance at the marginal tumor regions should be better characterized in future studies.

### Study Power Considerations

In this study, the prevalence of lesional samples was 84% (102 of 122) among all samples and 82% (80 of 97) among the glioma samples. The Buderer formula (34), with accuracy set at 0.05, prevalence of disease in the tested population set at 84%, and a sample size of 122, resulted in z=1.127 and confidence interval (CI) within 0.70 to 0.75 for sensitivity and z=1.687 and CI of 0.90 to 0.92 for specificity. For gliomas, the power analysis resulted in z=0.594 with CI of <50% for sensitivity and z=1.307 and CI of 0.80 to 0.85 for specificity. Although our study was underpowered for sensitivity, it is the first feasibility study to provide prevalence and diagnostic accuracy values for the planning of future diagnostic accuracy studies using CLE. For example, assuming 82% prevalence of tumor-positive samples, 94% specificity, and 66% sensitivity, the study size should be 419 for calculation of sensitivity and 103 for specificity to obtain a 95% CI and 0.05 accuracy.

### Study Limitations

Factors such as biopsy location, tumor type, the experience of the CLE user, and the extent of the scanned region of interest may have introduced biases and should be controlled for in future studies. This study is limited to *ex vivo* analyses because of the unavailability of the sterile CLE probe sheaths during the time the study was conducted; thus, human *in vivo* use was precluded. However, this situation may be viewed as advantageous because it may have decreased sampling error compared with *in vivo* acquisition for methodologic reasons, as it is more certain that the CLE images were obtained from and directly correlated with the same biopsy region, which is often difficult to accomplish intraoperatively.

Nevertheless, we acknowledge that sampling error is unavoidable with the small field of view of the imaging probe. H&E slides of multiple samples were inhomogeneous when viewed under the microscope: some had areas of necrosis encompassing up to 75% of the whole section, and some had infiltrating tumor in only 20% of their area, with the remaining regions consisting of vessels and hemorrhage. Use of CLE may identify and distinguish tumor cellular areas that are later missed on standard histologic examination (**Figure 4**). Two CLE optical biopsies that were classified as having normal results (i.e., false-negative results) had dark backgrounds and normal-appearing vessels suggestive of normal brain; however, few regions of interest were imaged with CLE for this sort of optical biopsy, and therefore the infiltrative tumor areas detected on H&E slides may have been missed. To minimize the sampling error, we moved the probe across specimens to visualize larger areas and to identify locations with high-quality confocal images bearing diagnostic features, but the area that is scanned is subject to user bias.

## Conclusion

Our results demonstrate the feasibility of incorporating CLE assessment of tissue microarchitecture in FGS. CLE optical biopsies showed high specificity and positive predictive value in fresh *ex vivo* glioma samples. These results reinforce the utility of CLE in providing real-time intraoperative cell resolution imaging to the neurosurgeon that is comparable to and faster than conventional histologic analysis from tissue biopsies in the operating room. These results also provide background data for planning future *in vivo* clinical studies.

## Data Availability Statement

The original contributions presented in the study are included in the article/supplementary material. Further inquiries can be directed to the corresponding author.

## Ethics Statement

The studies involving human participants were reviewed and approved by St. Joseph’s Hospital and Medical Center with approval from the Institutional Review Board for Human Research. The patients/participants provided their written informed consent to participate in this study.

## Author Contributions

Study planning and coordination: PN, MP, JE, EB. Acquisition of surgical biopsies and patient recruitment: PN. Acquisition of confocal images: EB, XZ. Processing and organizing of the data and confocal images: BN, DF, EB. Assessment of confocal images: EB, MP, VB, JE. Histology processing: EB. Histology reading: JE. Statistical analysis: EB, BN, DF. Writing a draft: BN, DF, EB. Review of the draft: EB, BN, DF, VB, MP, PN. All authors contributed to the article and approved the submitted version.

## Funding

This research received material support from Carl Zeiss Meditec, AG, and financial support from the Barrow Neurological Foundation and the Newsome Chair in Neurosurgery Research held by Dr. Preul.

## Conflict of Interest

The authors declare that the research was conducted in the absence of any commercial or financial relationships that could be construed as a conflict of interest.

## Abbreviations

5-ALA, 5-aminolevulinic acid; CI, confidence interval; CLE, confocal laser endomicroscope, confocal laser endomicroscopy; FDA, US Food and Drug Administration; FGS, fluorescence-guided surgery; FNa, fluorescein sodium; H&E, hematoxylin-eosin; HGG, high-grade glioma; ICG, indocyanine green.
